# Potential Impact of *DPYD* Variation on Fluoropyrimidine Drug Response in sub-Saharan African Populations

**DOI:** 10.3389/fgene.2021.626954

**Published:** 2021-03-09

**Authors:** Jorge E. B. da Rocha, Zané Lombard, Michèle Ramsay

**Affiliations:** ^1^Faculty of Health Sciences, Sydney Brenner Institute for Molecular Bioscience, University of the Witwatersrand, Johannesburg, South Africa; ^2^Division of Human Genetics, National Health Laboratory Service and School of Pathology, Faculty of Health Sciences, University of the Witwatersrand, Johannesburg, South Africa

**Keywords:** pharmacogenomics, *DPYD*, fluoropyrimidine drugs, cancer, precision medicine, Africa, precision medicine

## Abstract

Cancer is a critical health burden in Africa, and mortality rates are rising rapidly. Treatments are expensive and often cause adverse drug reactions (ADRs). Fluoropyrimidine treatments can lead to severe toxicity events which have been linked to variants within the dihydropyrimidine dehydrogenase (*DPYD*) gene. There are clinical guidelines to improve safety outcomes of treatment, but these are primarily based on variants assessed in non-African populations. Whole genome sequencing data from the 1000 Genomes Project and the African Genome Variation Project were mined to assess variation in *DPYD* in eight sub-Saharan African populations. Variant functional annotation was performed with a series of bioinformatics tools to assess potential likelihood of deleterious impact. There were 29 *DPYD* coding variants identified in the datasets assessed, of which 25 are rare, and some of which are known to be deleterious. One African-specific variant (rs115232898-C), is common in sub-Saharan Africans (1–4%) and known to reduce the function of the dihydropyrimidine dehydrogenase enzyme (DPD), having been linked to cases of severe toxicity. This variant, once validated in clinical trials, should be considered for inclusion in clinical guidelines for use in sub-Saharan African populations. The rs2297595-C variant is less well-characterized in terms of effect, but shows significant allele frequency differences between sub-Saharan African populations (0.5–11.5%; *p* = 1.5 × 10^−4^), and is more common in East African populations. This study highlights the relevance of African-data informed guidelines for fluorouracil drug safety in sub-Saharan Africans, and the need for region-specific data to ensure that Africans may benefit optimally from a precision medicine approach.

## 1. Introduction

Pharmacogenomics (PGx) combines expertise from the fields of pharmacology and human genetics in order to better understand the relationship between the individual, their genes, and their response to specific drugs (Pirmohamed, 2001). The outcomes of pharmacogenomic research contribute to precision medicine, to attain medical care that has high therapeutic efficacy while minimizing side-effects, informed by the presence of specific genetic variants. Although some pharmacogenomics research is taking place on the African continent, pharmacogenomics knowledge is more limited for African populations than for European or Asian populations (Radouani et al., 2020). Africa is facing a dual burden of disease from communicable and non-communicable diseases, and the costs incurred by adverse drug reactions (ADRs) during treatment can significantly add to such burdens (Dandara et al., 2019).

On a continent-wide level, cancer is the second most common non-communicable disease-related cause of death in Africa (WHO, 2014). Cancer is often severe, and treatments are expensive, with high risk of adverse effects. This can place a large financial burden on communities already struggling with poverty (Mayosi et al., 2009). Cancer rates are rising in Africa (WHO, 2013) and there is a need for new, advanced strategies for prevention, diagnosis and treatment across the continent. Cancer PGx guided by both somatic and germline variation can be used to improve treatment efficacy, and prevent ADRs. Chemotherapeutic agents are often cytotoxic and characterization of germline variants can be used to improve drug safety, although establishing such relationships often requires complex and expensive clinical trials (Wheeler et al., 2013).

The *DPYD* gene encodes dihydropyrimidine dehydrogenase (DPD), an enzyme involved in the metabolism of fluoropyrimidines, such as fluorouracil (5FU) (a WHO essential medicine), capecitabine and tegafur. Fluoropyrimidines are commonly used to treat a wide variety of cancers and exert their anti-cancer effect in multiple ways, but primarily by inhibition of the thymidylate synthase enzyme. *DPYD* variants that lower gene expression or produce non-active DPD can cause severe, life threatening side effects following treatment with standard doses of fluoropyrimidine based drugs (Loganayagam et al., 2013). Partial or complete deficiency of DPD is present in populations of European descent in approximately 4 and 0.2% respectively (Morel et al., 2006). Single nucleotide variants in *DPYD* have been linked to increased risk of severe toxicity following fluorouracil treatment in European (Saif, 2013) and Asian (Zhang et al., 2012) cancer patient cohorts, and a variant specific to African Americans (compared to European Americans) was found to reduce DPD activity (Offer et al., 2013).

Genotyping strategies have been used to identify patients at risk for toxic events and to ensure effective treatment by adjusting the dosage or suggesting an alternative treatment (Henricks et al., 2018). Current PGx strategies are guided by variants identified in studies of mainly European and Asian populations and do not take into account the potential effects of genetic variation in African populations. Known PGx variants are cataloged in large pharmacogenomic databases, such as PharmGKB (Whirl-Carrillo et al., 2012) and the Clinical Pharmacogenomics Implementation Consortium (Amstutz et al., 2018).

African whole genome sequence (WGS) data from two large studies provides the opportunity to mine data relevant to the pharmacogenomics impact of *DPYD* sequence variation in African populations. The first is the 1000 Genomes Project (KGP) (Auton et al., 2015), which produced WGS data for 26 global populations, including five from the African continent. The second is the African Genome Variation Project (Gurdasani et al., 2015) which characterized African genetic variation for populations across the continent, and has WGS data from 320 individuals from Ethiopia, South Africa, and Uganda.

This study aims to characterize *DPYD* variation from whole genome sequence data from sub-Saharan Africans to assess pharmacogenomic impact and to establish the potential risk for adverse drug reactions. *In silico* functional prediction was used to characterize variants with uncertain clinical significance. These analyses could help to identify variants associated with potential risk of using fluoropyrimidine treatments in African populations, and suggest their assessment for consideration in clinical guidelines in African populations.

## 2. Methodology

**Figure 1:**
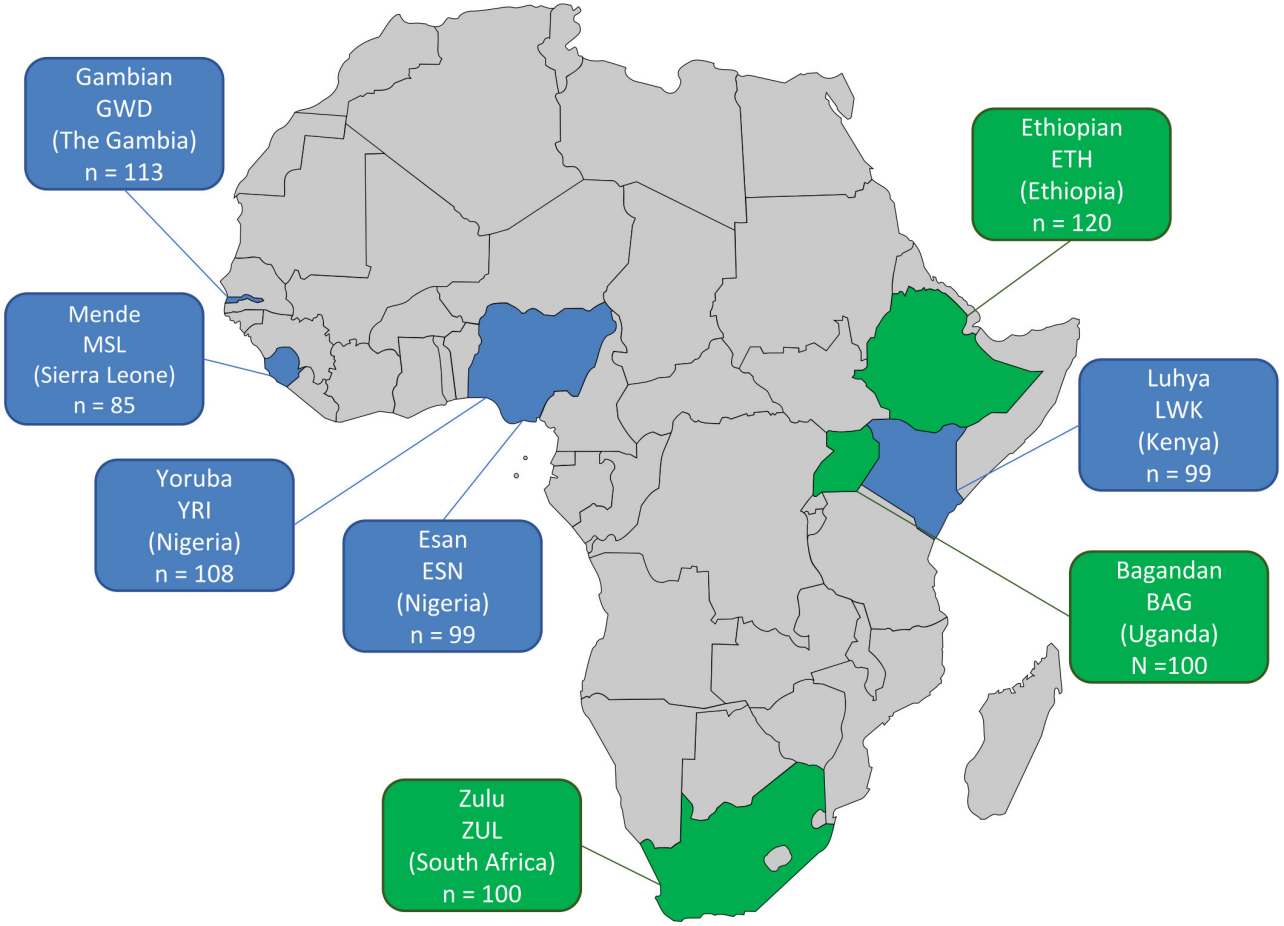
African population datasets included in this study. The country of origin, abbreviation of the population and number of samples with WGS data (*n*) are shown per population. KGP data—blue, 4X average coverage; AGVP data—green, 4-8X average coverage.

African WGS data from the KGP dataset was accessed for the following populations: Nigerian Yoruba (YRI) and Esan (ESN), Mende of Sierra Leone (MSL), Gambian (GWD), and Kenyan Luhya (LWK). Variant call files were downloaded from (ftp://ftp.1000genomes.ebi.ac.uk/vol1/ftp/phase3/data). The KGP WGS data has an average coverage of 4X (Auton et al., 2015) and the number of individuals sequenced per population is shown in Figure 1, for a total of 504 individuals. The KGP Gambian population includes individuals from the Fula, Jola, Woloff, and Mandinka ethnic groups. In addition, the following WGS data from the African Genome Variation Project (Gurdasani et al., 2015) were included: Ethiopian (ETH) (4-8x coverage), South African Zulu (ZUL) (4x), and Ugandan Bagandan (BAG) (4x) (European Genome-phenome Archive accession number EGAS00001000363 - with permission from AGVP data access committee). The AGVP Ethiopian population includes individuals from the Amhara, Oromo, Somali, Wolayta, and Gumuz ethic groups. As neither the KGP nor the AGVP included individuals of North African ancestry, this study focuses on 824 sub-Saharan Africans from these combined datasets. The diversity of countries and ethnic groups in this set provides a broad overview of sub-Saharan diversity.

The Clinical Pharmacogenetics Implementation Consortium (CPIC) (Amstutz et al., 2018) data on functional alleles for *DPYD* was obtained from https://cpicpgx.org/guidelines/guideline-for-fluoropyrimidines-and-dpyd/, (accessed 25/06/2020). It shows positions for known *DPYD* variants and functional classification of allele effect. Reference tables for nomenclature and positions were obtained from Pharmvar, https://www.pharmvar.org/gene/DPYD (accessed 25/06/2020). These datasets are based on published outcomes of variant effect from both *in vivo* and *in vitro* studies. Variant effects are classified as “No Function” for those that completely knockout gene function, “Reduced Function” for those that reduce DPD activity significantly, and “Normal” for enzymes with wild type activity levels (Amstutz et al., 2018).

Variant Effect Predictor (VEP, release v86) (McLaren et al., 2016) and SnpEff (v4.3t) (Cingolani et al., 2012) were used to annotate genomic variants extracted from WGS with information regarding their type, functional impact and location based on the GRCh37 reference genome. A series of additional plugins for variant functional prediction were included in VEP: MetaSVM, MetaLR, VEST, and FATHMM. These were selected based on their accuracy for prediction of protein impacting variation (Liu et al., 2016). Missense type variants (as classified by VEP and SnpEff) were selected for analysis, along with loss of function variant types (e.g., stop gain, splice acceptor). Other variant identifiers, such as dbSNP ID (Sherry et al., 2001), and COSMIC ID (Tate et al., 2019) were included in the annotation. Variant alleles were indicated by those in the forward direction as indicated by GRCh37 co-ordinate.

Allele frequencies were compared between African populations using a chi-squared test using R v3.6.3 (R Core Team, 2013). Continental African frequencies (Cont. AFR) are the combination of African populations from the KGP (excluding diaspora ASW and ACB) and the AGVP. Global population frequencies were obtained from the KGP superpopulations (grouped populations by greater geographic region) (Auton et al., 2015). In an attempt to quantify inter-African population differences for *DPYD*, Wright's fixation index (*F*_*ST*_) estimate was calculated with PLINK (Chang et al., 2015) for the entire *DPYD* gene region, based on 2566 markers with valid estimates. Valid *F*_*ST*_ estimates for only four of the selected missense and LoF variants are available due to low allele frequencies, and high missingness across the merged KGP and AGVP datasets.

## 3. Results

Most variants observed in the *DPYD* coding region were rare, with some displaying frequency differences between African populations. There were 29 coding variants (28 missense, 1 loss of function) identified in the *DPYD* gene in the WGS datasets assessed (Table 1). There were an additional 28 synonymous variants, and 12,528 intronic variants across the gene region for comparative analyses. In comparison to external datasets in Pharmvar or CPIC, there are 88 non-redundant *DPYD* coding variants recorded in these two datasets (aggregated from multiple global studies). The weighted *F*_*ST*_ estimate for the eight populations across the *DPYD* gene was 0.0103, and the mean estimate was 0.009.

**Table 1:** *DYPD* missense allele frequencies in different African populations based on WGS data.

**ID**	**GRCH37**	**GWD**	**MSL**	**ESN**	**YRI**	**LWK**	**ZUL**	**BAG**	**ETH**
rs114096998	1:97544543_G_**T**	0.0266	0.0588	0.0306	0.0421	0.0204	0.005	0.005	0.0000
rs139459586	1:97544632_A_**C**	0.0000	0.0000	0.0000	0.0093	0.0000	0.000	0.000	0.0000
rs201268750	1:97544676_G_**T**	0.0000	0.0000	0.0000	0.0046	0.0000	0.000	0.000	0.0000
rs145529148	1:97544695_T_**C**	0.0000	0.0000	0.0000	0.0000	0.0000	0.005	0.005	0.0000
rs371313778	1:97700416_C_**T**	0.0044	0.0000	0.0000	0.0000	0.0000	0.000	0.000	0.0000
rs547099198	1:97700472_G_**A**	0.0000	0.0000	0.0000	0.0000	0.0000	0.010	0.000	0.0000
rs60511679	1:97770919_A_**C**	0.0000	0.0000	0.0000	0.0093	0.0000	0.000	0.000	0.0000
rs1801160	1:97770920_C_**T**	0.0398	0.0118	0.0051	0.0139	0.0606	0.010	0.040	0.0667
rs145548112	1:97771751_C_**T**	0.0000	0.0000	0.0051	0.0000	0.0000	0.000	0.000	0.0000
COSM1688098	1:97847978_C_**A**	0.0000	0.0000	0.0000	0.0000	0.0000	0.000	0.000	0.0042
rs548783838	1:97847992_C_**T**	0.0000	0.0000	0.0000	0.0046	0.0000	0.000	0.000	0.0000
rs1801159	1:97981395_T_**C**	0.0796	0.0706	0.1869	0.1481	0.3030	0.185	0.225	0.1625
rs142619737	1:97981407_C_**T**	0.0088	0.0059	0.0000	0.0046	0.0000	0.000	0.000	0.0000
rs1801158	1:97981421_C_**T**	0.0000	0.0000	0.0000	0.0000	0.0000	0.000	0.000	0.0042
rs760663364	1:97981484_G_**A**	0.0000	0.0000	0.0000	0.0000	0.0000	0.000	0.000	0.0042
rs144395748	1:98015282_G_**C**	0.0088	0.0000	0.0000	0.0000	0.0000	0.000	0.000	0.0000
rs72975710	1:98015291_G_**A**	0.0000	0.0000	0.0051	0.0093	0.0051	0.000	0.005	0.0000
rs61622928	1:98039437_C_**T**	0.1106	0.0765	0.0657	0.0972	0.0556	0.075	0.125	0.1083
rs143815742	1:98039474_C_**A**	0.0044	0.0000	0.0000	0.0000	0.0000	0.000	0.000	0.0000
-	1:98039520_G_**A**	0.0000	0.0000	0.0000	0.0000	0.0000	0.000	0.000	0.0042
rs144935781	1:98039526_T_**C**	0.0000	0.0059	0.0000	0.0000	0.0000	0.000	0.000	0.0000
rs573299212	1:98058829_C_**T**	0.0000	0.0000	0.0000	0.0000	0.0000	0.010	0.000	0.0000
rs369575517	1:98058923_G_**A**	0.0000	0.0000	0.0000	0.0000	0.0000	0.045	0.000	0.0000
rs146356975	1:98060705_T_**C**	0.0088	0.0000	0.0000	0.0000	0.0000	0.000	0.005	0.0000
rs115232898	1:98165030_T_**C**	0.0398	0.0176	0.0101	0.0417	0.0152	0.030	0.020	0.0000
rs374531732	1:98165034_C_**T**	0.0000	0.0000	0.0000	0.0000	0.0000	0.005	0.000	0.0000
rs2297595	1:98165091_T_**C**	0.0266	0.0118	0.0051	0.0093	0.1010	0.115	0.065	0.0708
rs1801265	1:98348885_A_**G**	0.4646	0.3941	0.4293	0.4352	0.5000	0.615	0.480	0.3375

*Frequencies are those of the alternate allele (bolded)*.

Of the identified missense variants, 24 were rare or singleton variants. Due to low coverage, these rare alleles require confirmation via higher coverage sequencing. Five variants were found to be polymorphic in African populations: rs115232898-C, rs2297595-C, rs61622928-T rs12022243-T, and rs1801265-G. The rs115232898-C variant has a known deleterious impact on DPD function. Notably, other well-characterized deleterious *DPYD* variants (rs3918290-T, rs67376798-T, rs55886062-G, and rs75017182-G) (Froehlich et al., 2015) were not observed in the African population datasets assessed (Supplementary Table 1). *F*_*ST*_ estimates, shown in brackets, for the rs1801160-T (0.0143), rs1801159-C (0.033), rs2297595-C (0.033) variants are higher than gene wide estimates, while that of rs61622928-T (0.003) is lower. One loss of function singleton allele (rs72549310-A) was present in an individual from the Esan population.

**Figure 2:**
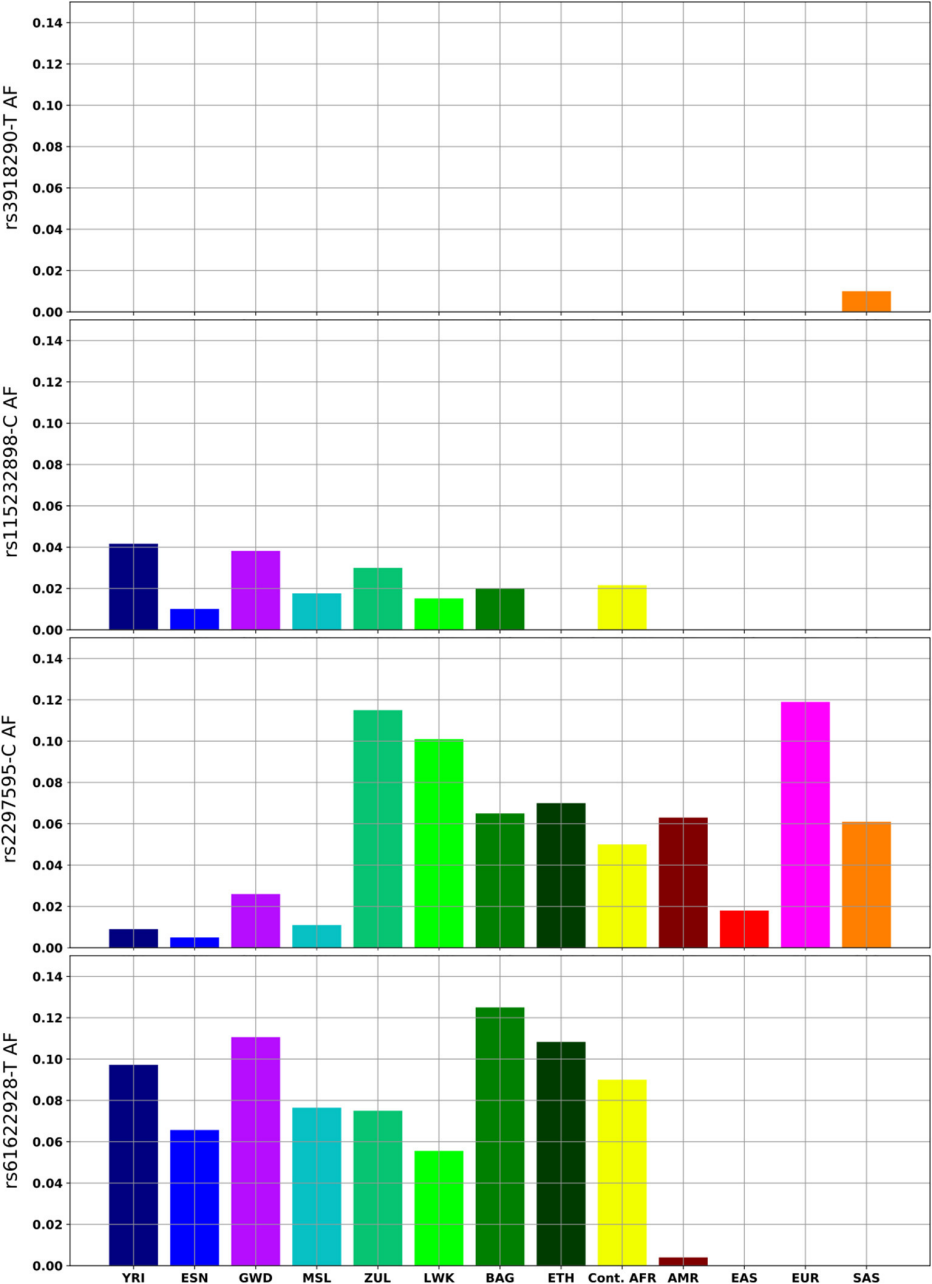
*DPYD* variants with clinical relevance or with notable frequency differences in African and global populations. Continental Africans (Cont.AFR); Admixed American (AMR); East Asian (EAS); European (EUR); South Asian (SAS).

The rs115232898-C variant is a known deleterious variant, which, although it does not completely knock out DPD function, does significantly reduce enzymatic activity (Offer et al., 2014). It is an African-specific variant, and ranges in frequency between 1-4% in African populations in which it is present (Figure 2). It was not observed in the Ethiopian sample. The rs3918290-T variant is rare in the KGP datasets and was only observed in the YRI (0.93%), however the CPIC datasets include additional studies where it is also found in Europeans (0.79%), African Americans/African Caribbeans (0.31%) and Latin Americans (0.08%) (Supplementary Table 1).

The rs2297595-C variant is present in all African populations, but significant frequency differences were observed between west and east (*p* = 1.5 × 10^−4^), and between west and southern Africans (*p* = 5.4 × 10^−6^). It is rare in west Africans ( 1%) while more common in the South African Zulu (12%) and east African (6–10%) population groups (Figure 2). The estimated *F*_*ST*_ value (0.033) is higher for this variant than the gene wide mean estimate (0.009), supporting higher population differentiation for this variant. The rs61622928 variant is an African-specific common variant with allele frequency differences across African populations (ranging from 5 to 12%), and not significantly different between African populations (*p* = 0.695). This is supported by its low estimated *F*_*ST*_ (0.003) relative to the gene wide mean.

The rs72549310-A stop gain variant was observed only in the Esan population, with only one heterozygote individual in the dataset. This variant has no current links to clinical findings, but has been shown to result in a non-functional transcript in mammalian expression systems (Offer et al., 2014).

Three rare African variants have evidence or prediction of functional impact (rs146356975-C rs144395748-C and COSM1688098-A). rs146356975 C was present in only two Gambian individuals and one Bagandan individual. There is *in vitro* evidence showing that this variant does significantly reduce DPD activity (Offer et al., 2014). rs144395748-C and COSM1688098-A are predicted to be deleterious by all four tools in the model used to predict variant effects. rs144395748-C is a doubleton, observed in two heterozygous Gambian individuals, while COSM1688098-A is a singleton observed only in the Ethiopian group.

Two variants in the previously described set (rs146356975-C and rs115232898-C) are listed within CPIC datasets as having evidence of deleterious or no function effect on DPD activity. There are 27 such variants in total listed in the CPIC datasets (Supplementary Table 1). Most are rare across the global populations for which genomic datasets are available. Of these, 21 are missense variants, of which 13 were predicted to have a deleterious impact on protein structure.

## 4. Discussion

Genetic variation in *DYPD* can affect fluoropyrimidine drug response during cancer treatment, and can lead to severe toxic reactions. This study examined WGS data to gain insights into *DYPD* variation in African populations. The data were mined for coding region variants, and two were previously annotated in CPIC and Pharmvar as having known functional consequences. Most of the identified variant alleles were rare and observed as single- or doubletons, thus requiring validation. However, three notable variants were found to be common in African populations and may be relevant to the outcomes of fluorouracil treatment and safety in Africans.

Fluorouracil toxicity has a wide range of symptoms which include vomiting, nausea and kidney failure. Pharmacogenomics-based clinical guidelines describe the dosage or recommended drug changes for individuals with heterozygous genotypes of deleterious variants. The rs3918290-T, rs67376798-T, rs55886062-G, and rs75017182-G variants have been shown to explain 20–30% of early onset fluorouracil related toxicities in European cancer patients (Froehlich et al., 2015). Early onset indicates that the toxic reaction occurs shortly after treatment is administered. As these variants have not been observed in African populations, they would have little relevance in Africa. Other variants which are common to African populations could, however, potentially contribute to the risk for fluoropyrimidine toxicity, if their predicted effects were confirmed in clinical trials.

The *DYPD* rs115232898-C variant appears to be African-specific with an average frequency of 3% (but not observed in the Ethiopian group) and is predicted likely to have a significant impact on the safety of fluorouracil treatment. It has also been observed in the KGP Puerto Rican population (<1%) who may have some African ancestry. The C/T genotype of rs115232898 has been significantly associated with fluorouracil toxicity in an Afro-Caribbean woman who presented with life-threatening toxic effects (Zaanan et al., 2014) and in an African-American patient also presenting with severe toxic effects (Saif et al., 2014). A functional study on the variant reported by Zaanan et al. (2014) found that enzyme activity was reduced by 29% (Offer and Diasio, 2014). The C/T genotype has also been associated with lower DPD activity in a cohort of healthy African-Americans exposed to fluorouracil (*n* = 94, *p* = 0.00027) (Offer et al., 2013). It is likely that these individuals have this variant as part of their African ancestral heritage, and the observed effects support the high confidence *in silico* predictions made by the toolsets used in this study. Together this evidence warrants the urgent need for studies in continental Africans currently being treated for cancer with fluorouracil, to validate the predicted effect in patients with the C/T genotype. Should it be confirmed, a genotype assay could be recommended in preventative guidelines to avoid toxicity.

The rs61622928-T variant has been noted in functional assays to decrease activity of DPD (Ezzeldin et al., 2005; Thomas et al., 2016), but other studies present no effect on activity linked to this variant (Kuilenburg et al., 2016). This variant is African specific, present in Africans at an average allele frequency of 9%, but absent in other global populations (Figure 2). Further research should aim to characterize the functional impact of this variant on PGx for Africans treated with fluorouracil.

The rs2297595-C variant is present in all African populations, but shows regional allele frequency differences, being rare in West Africa ( 1%), more common in East Africa (~8%) and with a frequency of 12% in the South African Zulu. Predicted to be likely functionally deleterious by all toolsets, the rs2297595 C/C and C/T genotypes have been associated with fluorouracil toxicity in European breast and gastroesophageal cancer patients (*n* = 92, *p* = 0.001) (Gross et al., 2008), European colorectal cancer patients (*n* = 568, *p* < 0.05) (Deenen et al., 2011), and Italian cancer patients (*n* = 64, *p* = 0.022) (Falvella et al., 2015). However, there have been conflicting reports showing no evidence of an association with either genotype with toxicity in a mixed population cohort (*n* = 430, *p* = 0.396) (Loganayagam et al., 2013), an Asian cohort of gastric cancer patients (*n* = 362, *p* = 0.4294) (Zhang et al., 2012), and a case/control analysis of a European cohort of gastric cancer patients (*n* = 410, 95 cases) (Toffoli et al., 2015). In light of the uncertain status of this variant, it is important to explore its effect in African patients, especially in East and South African populations, where the variant is more common.

One rare variant with known functional impact (rs146356975-C) and two with predicted functional impact (rs144395748-C and COSM1688098-A) were present in the African datasets. Although rare, their impact on treatment outcomes in African individuals should be explored, as they may provide further insights into the biological pathway to toxicity.

These findings show that there are *DPYD* variants in Africans that may affect fluorouracil treatment, and the frequency of these can be significantly different between African and non-African populations. Variants identified to explain a significant number of fluorouracil related toxicities (Froehlich et al., 2015) (rs3918290, rs67376798, rs55886062, rs75017182) in other populations were not observed in the African datasets assessed. As the rs115232898-C variant is common in Africans, and has functional impact, it may be a valuable addition to the four variants above while evaluating risk of toxicity in African populations. Guidelines should also consider inter-African population differences. For example, rs115232898-C was not detected in the Ethiopian population assessed, and would therefore have limited use in that group. Although we were limited in our assessment of inter-African population differences across the entire *DPYD* gene, future work should include additional African samples (with high coverage sequencing) which are jointly called.

A limitation of this work is that the populations and ethnic groups assessed do not fully represent diversity across the African continent, highlighting the importance of greater sampling of African populations to characterize a more complete landscape of genetic diversity. Another limitation is that the AGVP and KGP datasets used were not jointly called and this led to high missingness of variants in the combined dataset and reduced the number of variants for which *F*_*ST*_ estimates could be calculated.

As cancer rates continue to rise in Africa, it is important to consider the pharmacogenomic risk of using fluoropyrimidine based treatments in African populations. Variants with high predictive value for early onset toxicity in Europeans were not observed in the African populations studied. African *DPYD* variants with predicted functional impact, such as rs115232898-C, should be validated and considered for inclusion in guidelines or testing strategies for African populations. The rs61622928-T variant is specific and common in African populations, and therefore requires functional characterization in African populations. The rs2297595-C variant is common in African and European populations, but functional interpretations are not conclusive. Assessing the impact of the rs2297595-C variant would be useful for guidelines for East Africans, where this variant is more common. These common *DPYD* variants, in addition to numerous rare population specific variants pose challenges for implementation of safety and testing strategies for African individuals. Clinical trials of fluorouracil treatment in Africans are needed to establish which variants are most informative in the context of PGx. This knowledge, in combination with allele frequency data, could lead to the development of precision public health guidance of key cancer treatments for African populations.

## Data Availability Statement

The 1,000 Genomes Project datasets used in this study can be found at https://www.internationalgenome.org/data/. The AGVP datasets are available through the European Genome-phenome Archive (EGA)-accession number EGAS00001000363-with permission from AGVP data access committee.

## Author Contributions

All authors contributed to concept and study design, data analysis, and drafting of the manuscript. ZL and MR performed critical revision of the manuscript, and provided supervision.

## Conflict of Interest

The authors declare that the research was conducted in the absence of any commercial or financial relationships that could be construed as a potential conflict of interest.
