# Supplementary material for: Potential Impact of DPYD Variation on Fluoropyrimidine Drug Response in sub-Saharan African Populations
Source: Front Genet. 2021 Mar 9;12:626954. doi: 10.3389/fgene.2021.626954 (PMC7985174; doi:10.3389/fgene.2021.626954)
Supplement: Supplementary file 1 [file Data_Sheet_1.PDF]

frontiers

Supplementary Material

1 SUPPLEMENTARY TABLES AND FIGURES

The Clinical Pharmacogenetics Implementation Consortium (CPIC) data on functional alleles for *DPYD* was obtained from https://cpicpgx.org/guidelines/guideline-for-fluoropyrimidines-and-dpyd/, (accessed 25/06/2020). Frequencies displayed in Table S1 are as captured by CPIC from the 1000 Genomes Project, Exac, and other projects focused on *DPYD* variation alone.

1.1 Supplementary Tables

Table S1. CPIC known non-functional/reduced function allele frequencies

ID	cDNA	StarAllele	Functional Status	AA/AC	AM	C/SA	EA	EU	LA	NE	OCA	SSA
rs3918290	1905+1G>A	(*2A)	No function	0.0031	0	0.0051	0	0.0079	0.0008	0	0	0
rs72549303	1898delC	(*3)	No function	0	0	0	0	0	0	0	0	0
rs72549309	295_298delTCAT	(*7)	No function	0	0	0	0	0.0002	0.0001	0	0	0
rs1801266	703C>T	(*8)	No function	0	0	0.0002	0	0.0001	0	0	0	0
rs1801268	2983G>T	(*10)	No function	0	0	0	0	0	0	0	0	0
rs78060119	1156G>T	(*12)	No function	0	0	0	0	0	0	0	0	0
rs55886062.1	1679T>G	(*13)	No function	0	0	0	0	0.0006	0	0	0	0
Hap B3	1129-5923C>G + 1236G>A		Decreased function	0.0031	-	0.0197	0.0000	0.0237	0.0059	-	-	0.0000
rs67376798	2846A>T	-	Decreased function	0.0031	0	0.0006	0	0.0037	0.0021	0	0	0
rs115232898	557A>G	-	Decreased function	0.0123	0	0	0	0.0001	0.0012	0	0	0.0259
rs72549310	61C>T	-	No function	-	-	-	-	-	-	-	-	-
rs72549308	601A>C	-	No function	0	0	0	0	0	0	0	0	0
rs72549307	632A>G	-	No function	0	0	0	0	0	0	0	0	0
rs146356975	868A>G	-	Decreased function	0	0	0	0	0	0.0003	0	0	0.002
rs183385770	1024G>A	-	No function	0.0031	0	0	0	0	0	0	0	0
rs143154602	1057C>T	-	No function	0	0	0	0	0	0	0	0	0
rs186169810	1314T>G	-	Decreased function	0	0	0	0.0007	0	0	0	0	0
rs72549304	1475C>T	-	No function	0	0	0.0002	0	0	0	0	0	0
rs111858276	1484A>G	-	No function	0	0	0	0	0	0	0	0	0
rs138616379	1775G>A	-	No function	0	0	0	0	0	0	0	0	0
rs59086055	1774C>T	-	No function	0	0	0	0.0016	0	0	0	0	0
rs145773863	1777G>A	-	No function	0	0	0	0	0	0	0	0	0
rs137999090	2021G>A	-	No function	0	0	0	0	0	0	0	0	0
rs112766203.1	2279C>T	-	Decreased function	0	0	0.006	0	0	0	0	0	0
rs55674432	2639G>T	-	No function	0	0	0.0007	0	0	0	0	0	0
rs141044036	2872A>G	-	No function	0	0	0	0	0	0	0	0	0
rs72547601	2933A>G	-	No function	0	0	0	0	0	0	0	0	0

Hap B3 - rs75017182 and rs56038477

Population groupings as defined in CPIC tables. AA/AC - African American/African Caribbean, AM - American, C/SA - Central South Asian, EA - East Asian, EU - European, LA - Latin American, NE - Near Eastern, OCA - Oceanian, SSA - Sub-Saharan Africa.
